# Automotive Diesel Fuel Internal Stability Testing with the Use of UV and Temperature as Degradation Factors

**DOI:** 10.3390/ma15238548

**Published:** 2022-11-30

**Authors:** Michal Borecki, Mateusz Geca, Michael L. Korwin-Pawlowski

**Affiliations:** 1 Institute of Microelectronics and Optoelectronics, Warsaw University of Technology, 00-661 Warsaw, Poland; 2 Department of Electronics and Information Technology, Lublin University of Technology, 20-618 Lublin, Poland; 3 Département d’informatique et d’ingénierie, Université du Québec en Outaouais, Gatineau, QC J8X 3X7, Canada

**Keywords:** diesel fuel, fuel stability, internal stability, photostability, thermal stability, PAH

## Abstract

Diesel fuel stability can be considered from many points of view, of which the two considered most important are stability in contact with the environment and internal stability. Fuel stability in touch with the environment is often defined as oxidation stability, of which measurement procedures are well developed. The presented paper shows that fuel’s internal stability can also be important. The internal stability of diesel fuel with the local use of thermal and ultraviolet radiation (UV) as degradation factors and fluorescence signals as a probe is presented in this paper. We show that the internal degradation of fuel with temperature use differs from that with UV and simultaneous both factors use. Our study shows that using temperature as a degradation factor introduces significant fluorescence fading. Moreover, the fluorescence signal restores significantly later than the sample stabilizes at room temperature. The novelty proposed based on examination is hybrid degradation and an examination cycle that enables the simultaneous use of degradation factors and fluorescence reading. For this purpose, a dedicated measurement setup of signal control and processing was constructed and programmed. The measurement procedure of the data series for specific wavelength enables calculation of signal shifts that allow the internal stability classification of diesel fuel samples in less than 30 min with the cost of a single disposable capillary probe and one polymer plug. Premium and regular fuel examination results show that internal fuel stability can be related to polycyclic aromatic hydrocarbons (PAH) concentrations and can be modified with dedicated additives.

## 1. Introduction

For users of automotive diesel (EN 590), the stability of its parameters is a crucial issue [1]. Fuel stability may be analyzed from a producer, distribution chain, and end-user point of view and is directly connected with its composition. Primary chemical stability refers to the resistance of a chemical to change in a chemical reaction. While diesel fuel is a complex chemical system, reactions may occur inside the sample and in contact with surroundings, notably, tank walls and the atmosphere at specific conditions. The mentioned conditions, such as temperature and lighting, can be the energy source for the activation or progress of the fuel degradation reaction [2]. The standard diesel fuel stability testing includes oxidation stability affected by the contact of fuel with its environment. Internal stability refers to the chemical reactions inside the fuel exclusively between the internal components of the fuel. The internal stability of diesel fuel is not yet well recognized. Some initial examinations of the internal stability of fuels used infrared spectral characteristics comparisons [3].

Automotive diesel fuel consists of the fuel base, fuel improvers, and impurities. Standard fuel base components may include petroleum diesel (petro-diesel) and synthetic diesel (syn-diesel). According to the EN 590:2009 standard, the optional automotive diesel fuel components can consist of up to 7% of fatty acid methyl esters (FAME).

Petro-diesel is produced in a refinery [4]. Petro-diesel for road vehicles consists mainly of hydrocarbons with carbon numbers in the range of C9–C20, which result from distillation in the temperature range of 160–360 °C. It contains primarily branched-chain alkanes and alkenes, including cycloalkanes. Alkanes are only weakly reactive with most chemical compounds. Cycloalkanes in petro-diesel are very stable; their reactions are like alkanes [5]. Petro-diesel also contains different volumes of polycyclic aromatic hydrocarbons (PAHs), including oil-characteristic alkylated C2–C4-PAH [6,7,8]. The presence of PAHs in petro-diesel depends on the crude oil characteristics, the specific boiling point of PAH, the distillation range, and the use of a side-stream product of the distillation [9,10], as presented in Table 1. Determining the PAH concentration is not straightforward [11,12,13,14], but some PAHs offer exciting properties. For example, 1-methylnaphthalene, which delays long ignition, enables the use of it as the lowest reference point of cetane number (0) [15].

The stability of PAHs in free form can be deducted from recommended storage conditions. They should be kept in a tightly closed container kept at room temperature. For example, naphthalene sublimes at room temperature. However, the rest of the listed PAHs are stable under standard laboratory conditions, but all PAHs can be oxidized [16]. Some PAHs, such as perylene and benzo[a]pyrene, undergo photo-oxidation after sun or ultraviolet light irradiation. PAHs have intrinsic fluorescence properties in the ultraviolet and blue light spectrum [17]. Pyrene can act as an efficient fluorescent probe of polar solutions. The changes in the polarity of the immediate environment of the pyrene can result in the fluorescence spectrum changes of intensity or shift wavelength of peak [18,19]. Fluorescent sensitivity of pyrene to lipids was reported, with peaks of emissions observed at 375 nm, 400 nm, and a hill at 460 nm [20]. PAHs’ presence in diesel fuel is of concern because of their chemical activity and toxic potential [21,22,23].

Syn-diesel can be produced from any carbonaceous material based on pyrolysis, hydrotreatment, or gas-to-liquid conversion [24,25]. Syn-diesel manufactured from natural gas can create the best clean fuel characterized by a high level of stability [26,27].

Bio-diesel components may include fatty acid methyl esters (FAME), hydrogenated vegetable oil (HVO), or raw vegetable oil [28,29]. FAME may include saturated and unsaturated components [30]. Some unsaturated parts of fatty acids with double bonds are susceptible to chemical oxidation and can generate acids and water [31]. It should be noted that FAME prepared by transesterification oxidation stability can be four times worse than petro-diesel [32]. HVO derived from hydrogenation and hydrocracking has similar chemical properties as petro-diesel. Still, since it is free from sulfur, oxygen, and aromatic hydrocarbons, it is considered a high-quality diesel substitute and can be used directly as diesel fuel [33].

Diesel fuel often contains additives. Detergents and dispersants are the most crucial performance additives [34]. The function of other additives can be described as oxidation inhibitors (antioxidants), corrosion inhibitors, demulsifiers, anti-icing, and lubricants [35,36].

Diesel fuel may contain contaminations, such as water, waxes, microbes, and particulate matter [37,38]. Diesel fuel contamination starts at the refinery, continues at distribution and storage facilities, and ends at fuel tanks in vehicles. The particulate matter contaminants may include metals, such as Fe, Al, Cr, Cu, trace amounts of Zn, Na, Pb, metal oxides, debris, and oxidation by-products [39,40]. The fuel cleanliness characterization method was standardized in ISO 4406.

### 1.1. Automotive Diesel Fuel Stability

Automotive diesel fuel is a liquid mixture of liquids and particle components. This mixture’s main components are chemically stable hydrocarbons, but some active components, such as FAME and PAHs, are allowed in limited concentrations [41]. The basic properties of diesel fuel are described by three main characteristics of their hydrocarbon molecules, type-length, and shape of the hydrocarbon skeleton and functional groups present [42]. However, intermolecular interactions in diesel fuels are strong and cannot simply be neglected [43,44,45]. Thus, chemical and physical phenomena define the stability of automotive diesel fuel.

In everyday language, a chemical substance is stable if it does not react with the environment and is not internally self-degradable. In particular, the expected usefulness should be retained under standard storage conditions, including air, moisture, and exposure to temperature and light. Chemical stability, when used in the technical sense, means the thermodynamic stability of a chemical system. Thus, chemical stability can be defined in closed chemical systems, referred to as internal stability, or in open systems that are in contact with the environment [46,47].

The typical reactions that occur during diesel fuel storage, caused by contact with the environment, are hydrolysis, microbial growth, and oxidation. Hydrolysis happens when the fuel is exposed to water that has entered the tank with air, a situation described by relative humidity. The visible results of diesel fuel hydrolysis are a change in fuel coloration and sludge present at the bottom of the tank [48]. Microbes live in the water–diesel interface [49,50]. Oxidation reaction occurs when diesel fuel is exposed to oxygen, which happens in standard storage conditions. Oxygen attacks molecules of the fuel and various oxidation products are created, such as aldehydes, ketones, carboxylic acids, and insoluble deposits [51].

Impurities from the refining process, as well as from the bio-diesel components treatment, result in the degradation of the fuel [52,53,54]. Mixing of fuel components can lead to chemical reactions that result in undesired products [55]. By contrast, modern microemulsion fuels with excellent time stability are candidates as alternative fuels [56].

Physical fuel stability is often related to the colloid (emulsion) form of liquid that results in phase separation and sedimentation processes. The proposed reaction type that results in sediment is due to phenalene (PAH) converted to phenalenone, which reacts further [57]. Photophysical properties of phenalenone derivatives are that some absorb 300–429 nm and emit 348–578 nm of light [58]. Physical effects of colloid stability as phase separation are used in some standardized tests of fuel oxidation stability, for example, ISO 12205:1995, DIN EN 15751, and DIN EN 16091. In most examinations, oxygen is mixed with fuel, and temperature is elevated. Therefore, these tests do not represent real-life conditions, and in many cases, test results correlation to real-life usefulness is unclear [59]. Tests without aggressive fuel mixing with oxygen have been proposed, such as ASTM D4625. The result of such tests during which fuel samples are stored at elevated temperatures are available only after 24 weeks.

Physical parameters of colloids, including phase stability, can be examined with the use of an optical sensing method, such as UV-VIS, spectroscopy, turbidimetry, and dynamic light scattering [60,61,62,63]. Besides the physical phase of colloid stability, the thermo-stability and photostability of diesel fuel are under investigation [64]. The examination method of photostability is at the proposition phase [65].

### 1.2. Fuel Degradation Factors

The degradation factors can be of chemical and physical type. The most intuitive chemical degradation factor is the presence of oxygen. Still, its volume is limited as modern automotive fuel systems, and storage tanks are effectively sealed to limit evaporative emissions. Therefore, oxidation of fuel is possible with oxygen present in the tank. Assuming that a 10-L tank is half filled with fuel, we obtain 5 L of fuel and about 1 L of oxygen. Thus, 5 L of fuel with a density of 0.85 kg/L gives 4.25 kg. Assuming cetane as a representative hydrocarbon with 226.44 g/mol, we obtain 18.76 mols of fuel particles. Considering that 25% of fuel particles are chemically active, the result is 4.69 mols of active particles. Parameters for oxygen are density 1.429 kg/m^3^ and 32 g/mol; thus, we obtained 0.044 mols of oxygen. The conclusion of comparing the number of mols of oxygen and the unsaturated hydrocarbons is that oxygen can react with about 1% of fuel hydrocarbon components. Thus, direct fuel oxidation with an unlimited oxygen supply is just a model-type measure of an automotive diesel fuel’s actual stability.

Internal chemical contaminations can significantly impact fuel stability; thus, they can be considered indirect degradation factors. Activating reactions of contaminations with unsaturated hydrocarbon components sometimes require energy, and the energy type can be viewed as a degradation factor [66,67].

The most intuitive physical degradation factors are UV radiation and temperature.

It was shown that UV irradiation impacts diesel fuel’s chemical composition and selected physicochemical properties [68]. The temperature influence on diesel fuel degradation was also presented [69]. Since steel tanks are now being replaced with polymer tanks with visible transparency, the photostability of fuel gains significance.

### 1.3. Summary

Automotive diesel fuel may be described as a heterogeneous reactive mixture with the chemical stability of a kinematic and a thermodynamic type [70]. Molecules of automotive diesel fuel react not only with atmospheric oxygen but also with themselves. Moreover, some contaminants can act as catalysts for reactions [71]. Until now, fuel degradation is typically examined using standard glass containers in batch thermal stressing conditions. The typical sign of fuel degradation is the formation of solid deposits. In laboratories, internal fuel stability is characterized by decomposition products that are measured using gas chromatography and mass detection. In this method, the fuel sample usually requires initial preparation, including storage in specified conditions. Direct examinations of active fuel component concentrations that can affect internal fuel stability, such as PAH in non-water liquids, are also conducted in laboratory setups using glass cuvettes, specialized optoelectronic devices, and advanced chemical procedures [72,73]. By contrast, the ultraviolet-induced fluorescence signatures seem promising to evaluate diesel fuel for detecting PAH concentration. Reported results for standard examinations showed a peak for fluorescence excitation of about 370 nm and emission in the 420 nm range [74]. The fluorescence intensity versus pollution concentration first increased and then decreased.

As of today, used automotive fuel stability testing standards are not suitable for implementation in sensors, and a new method is required. While methods of oxidation stability are well described, the practices of fuel internal stability testing are in the initial stage of investigations. To examine the internal stability of fuel, the realization of reproducible and fast energy transfer methods to fuel samples is essential. Such energy transfer can be performed with the light irradiation of fuel closed in the transparent and thin walls of a capillary positioned over micro-heaters [75]. A similar configuration of the measurement system with remote local fuel heating, but without UV irradiation, was used to test fuel quality [76]. Additionally, a similar configuration measurement setup with UV irradiation of the fuel sample but without local heating was used to examine fuel sample degradation [77,78].

This paper presents new results of examinations of the internal stability of automobile diesel fuels with the simultaneous use of two degradation mechanisms: thermal and UV. The correct simultaneous application of degradation factors and fluorescence reading is not a trivial task since, during intensive heating and lighting of the sample, partial and temporary fading of the fluorescent signal informing about the state of the fuel is to be expected. In addition, the requirement of the time necessary to regenerate the signal is presented. The novelty of the proposed method is also in the construction of a measuring system that enables quick changes in the fuel state and synchronization of degrading factors with the test signals that induce fluorescence at the appropriate moments.

The paper is organized as follows: Section 2 describes the fuels used for the examination. Section 3 depicts a measurement head with a disposable optrode for automotive diesel fuel internal stability examination with the use of UV and temperature as the only degradation factors. Section 4 describes the measurement setup configuration, construction, and initial tests that are necessary to set the basic parameters of degradation cycles. Section 5 addresses operational research of degradation and examination cycles, including constant and modulated degradation factors. Section 6 reports the results of the fluorescent signal analysis in the hybrid degradation and examination cycle. Section 7 presents short conclusions.

## 2. Diesel Fuels for Experiments

**Figure 1:**
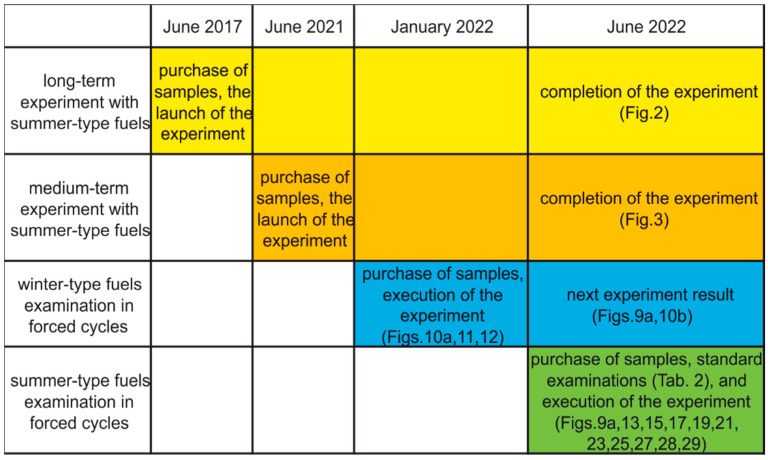
Gantt simplified chart of fuels examinations.

Samples of commercial, automotive diesel fuels that meet EU standards from the same manufacturer of regular and premium quality fuel were used in this study. The time sequence of the investigations is presented in Figure 1.

**Figure 2:**
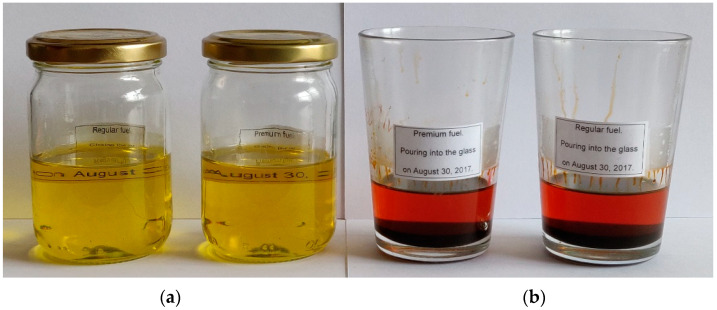
Premium and regular fuels stored in an optical darkroom for five years: (**a**) in closed jars; (**b**) in open glasses.

Premium fuels of summer type were subjected to a long-term degradation test in the optical darkroom, Figure 2.

The fuels were poured to the same level and stored in closed jars and opened glasses. Sealed fuels seem not to be degraded. Their color, as well as transparency, are unchanged. Samples with free access to the atmosphere broke down completely. Color changes and deposits are visible. Some of the fuel evaporated. Interestingly, it seems that the effect of long-term oxidation does not depend on the type of fuel tested.

According to the manufacturer, the main difference between summer and winter types of fuels was in used additives that change the structure of paraffin crystals, and thus the temperature blocking the cold filter (CFPP). The parameters of the fuels in the summer version and corresponding measurement standards are presented in Table 2. The difference between regular and premium versions is mainly in the cetane number (CN) and the biofuel content; the fuel stability measured according to EN15751 standard seems to be the same for both.

The fuel samples were used unmodified and modified with an additional commercial multipurpose enriching additive, which extends engine life by cleaning and protecting against sludge formation in the fuel line. This additive was selected on the assumption that sludge formation is one of the leading indicators of fuel degradation. It is, however, challenging to choose the additive for a particular diesel fuel to achieve the expected fuel stability [79]. The standard volume of fuel samples was 100 mL, while the additive capacity was 1 mL. The colors of the fuel sample and the additive were similar. The fuel samples for the experiment were positioned in measuring glasses 20 cm tall, with a scale, and stored in dark conditions at 20 °C. The following abbreviations of fuel samples are used further on: PDFU—premium diesel fuel unmodified, PDFA—premium diesel fuel with an additional enriching additive, RDFU—regular diesel fuel unmodified, and RDFA—regular diesel fuel with an enriching additive. To account for possible differences due to the season when the tests were made, RDFU(s) is regular diesel fuel used in summer, and RDFU(w) is used in winter. Besides the mentioned fuels in selected initial experiments, an old type of diesel fuel, 100% of FAME clear component, and reference diesel fuel obtained directly from a refinery were used.

## 3. Design Principle of the Head with Disposable Optrode for Diesel Fuel Internal Stability Examination with the Use of UV and Temperature as Exclusive Factors of Degradation

**Figure 3:**
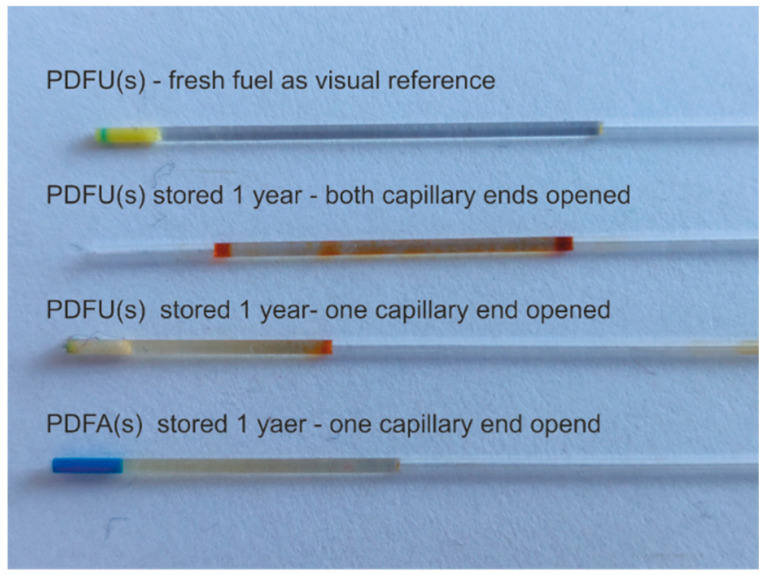
Results of diesel fuels of premium type PDFU(s) and PDFA(s) degradation positioned in a capillary placed at a windowsill for one year.

The proposed method of stability testing based on the observation of results of diesel fuel degradation in a capillary stored on the lab windowsill and exposed to variable temperature and lighting for one year is illustrated in Figure 3. The unmodified and modified fuels of regular type were examined in different conditions of contact with air. The sample at the top of Figure 3 of fresh fuel is transparent; on its left side, the polymer cork is visible. Below, premium fuel in unmodified type stored in a capillary with both ends open is presented. The most significant changes in fuel appearance are in the area of fuel contact with air, but inside the sample, changes are also visible. The piece of unmodified fuel with one end of the capillary closed also shows that most of the changes in fuel happen at its contact with air. The modified fuel with multipurpose enriching additive shows minor optical changes at air contact. Still, changes are present in the entire sample volume, indicating that the presence of surfactants prevents the fuel from directly contacting the air.

The photodegradation of fuel samples at air contact can be explained as the result of UV radiation absorption by a PAH molecule that is subsequently transferred to molecular oxygen dissolved from the air. This process generates highly reactive singlet oxygen atoms, which results in a series of specific reactions and degradation products. These products are not characteristic of a diesel fuel’s internal stability. As PAH inside a sample absorbs UV energy, it can also be transferred to other molecules that form free radicals that can join other PAH molecules forming substituted aromatics and causing subsequent formation and growth of other PAHs. Besides basic PAHs, alkenes and complex PAHs, such as 2-methylnaphthalene or butylbenzene, can react under UV radiation, influencing fluorescence. Thus, PAHs and alkenes may be a reason for sediment growth in the fuel sample, as presented in Figure 2. Therefore, fluorescence must be measured in bands, not peaks referring to separate specified PAHs. It is also expected that temperature is essential in affecting both the structure and diversity of the PAHs formed [80].

**Figure 4:**
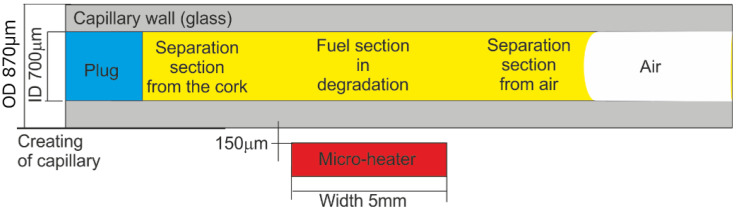
The idea of diesel fuel local section exclusive degradation with heat.

It is evident that internal fuel degradation is a natural phenomenon and cannot be omitted when fuel stability is examined. The task of internal stability examination can be realized with a capillary optrode that provides protection of a section of the fuel sample from direct contact with the environment but enables fuel sample local degradation with UV and thermal radiation. The idea of diesel fuel internal section exclusive degradation with heat is presented in Figure 4.

Capillaries used as optrodes were of the CV7087 type made from clear fused quartz by VitroCom, with lengths of 100 mm. The examined fuel sample was positioned in the capillary with the plug and by capillary forces. The fuel inside the capillary can be ordered into three sections, including two separation sections and a section under local degradation. The heat from the micro-heater concentrates in its close area [81]. The degradation temperature was set to 90 °C below the water boiling point and significantly higher than ambient air temperatures.

**Figure 5:**
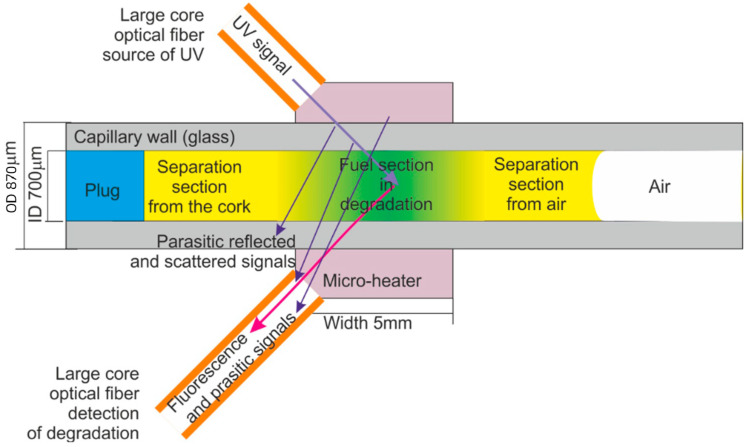
The idea of diesel fuel local section exclusive degradation with UV radiation and reading of degradation results.

The idea of diesel fuel internal degradation with UV radiation and the reading of degradation results is presented in Figure 4. The simplified scheme of the positions of light beam paths in the capillary head is illustrated in Figure 5. The main simplification is a two-dimensional and cut-down presentation of the structure of the head. The minor simplification assumes a low thickness of the capillary wall compared to the diameter of the capillary hole. Results are an arbitrary position of parasitic signals, which can be reflected from the capillary optrode, the micro-heater, and the optrode housing. The signal gathered in the detection fiber includes some portion of the excitation signal, but the usable signal of fluorescence is free of such influence. Therefore, the detection of fuel degradation can be appropriately performed.

The UV degradation factor must consider the Earth’s surface radiation by the sun [82]. That means that the deepest possible UV wavelength is 350 s. By contrast, used wavelengths should excite particles in diesel fuel, including particles of PAH and microbial organisms, giving a fluorescence signal. The wavelength of 365 nm was proposed for the excitation of PAH particles in the soil as anthracene and pyrene. This excitation results in emission peaks in 401 nm and 500 nm in bands wider than 50 nm [83]. For the mentioned excitation of fluorescence and degradation wavelength, it was shown that degradation of fuel results in a shift of peaks’ amplitude and wavelength as increasing peaks with greater wavelength and decreasing heights with lower wavelength. The 365 nm wavelength also excites the fluorescence of microbial organisms at 675 nm with a bandwidth of 10 nm.

## 4. Measurement Setup Construction and Configuration

**Figure 6:**
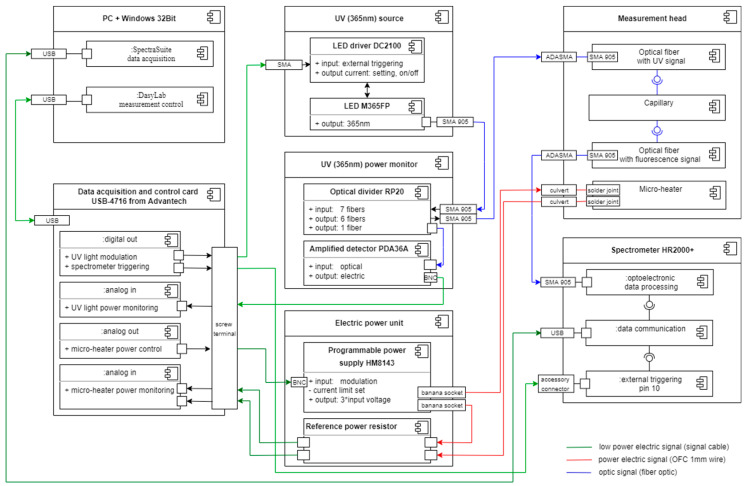
The component diagram of the measurement setup.

The measurement setup is built using standard commercial optoelectronic modules and a house-made head; it is presented in Figure 6.

The personal computer (PC) controls the experiments and data collection. The DasyLab software is used to control the degradation factors and data synchronization. The SpectraSuite software is for spectral data acquisition from Ocean Optics spectrometer HR2000+. DasyLab and SpectraSuite run simultaneously. DasyLab is equipped with a dedicated multipurpose script named DMPS. DasyLab measurement control is realized in hardware with the use of card USB-4716 from Advantech.

The LED M365FP and LED driver DC2100 were used as the UV (365 nm) source. The LED current limit was set to 50 mA. The measurement system controls the operation of the UV source directly with a triggering signal and monitors it with the amplified detector PDA36A and with a fiber bundle probe RP20 use. The fiber bundle probe consists of an arm to carry light from a LED source to a head and an arm to bring light to the detector. The optical power delivered by fiber to the head was 1.6 mW.

The optical signal is transmitted from the head to the HR2000+ spectrometer with the patch cord P600-1-UV-VIS. The DMPS also controls the spectrometer and SpectraSuite software’s work by triggering the acquisition signal. The integration time of spectra data is set in the software to 50 ms.

The DMPS is also equipped with a proportional–integral–derivative regulation section. It controls the micro-heater through the electric power unit consisting of the programmable power supply type HM8143 and the reference power resistor that acts as a current probe enabling the stabilization of temperature generated at the micro-heater’s surface to 90 ± 0.5 °C. The temperature of the fuel sample in the capillary above the micro-heater stabilizes in about 15 s.

**Figure 7:**
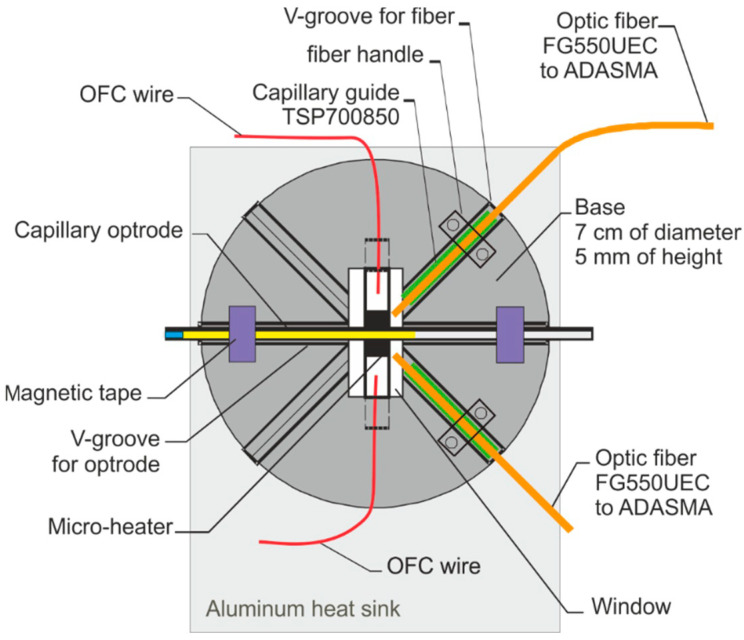
The schematic of the head.

**Figure 8:**
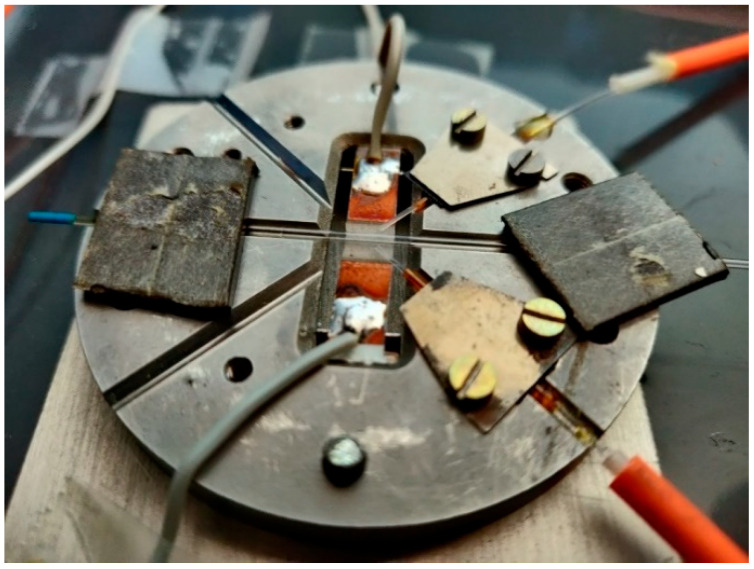
The view of house-made head.

The schematic of the head is presented in Figure 7, and the view of the house-made head is in Figure 8.

The base of the sensor head is made of alumina, its diameter is 7 cm, and its height is 5 mm. V-groves with uniform cross-sections were used to position the optical fibers and the optrode on the base. Capillary sections used as optrode were CV7087 from VitroCom, with an outer diameter of 870 µm. As the optical fiber outer diameter must be as close as possible to the capillary optrode, thus the optical fiber FG550UEC was used. Fiber buffers were removed for positioning purposes. To secure the fibers in the base and enable appropriate optical signal coupling, a short section of capillary TSP700850 from Polymicro with an outer diameter of 850 µm and inner diameter of 700 µm was used as a fiber’s buffer replacement. The head base was mounted on the aluminum heat sink, with triple the area of the base with 2 mm high copper spacers that enable the movement of air in the head. The head was mounted in a black plastic box 14 cm in height, 26 cm wide, and 45 cm long, with a removable cover, SMA fiber connectors, and culverts for power wires.

### 4.1. Initial Examinations with Static Fluorescence Readings

Preliminary tests determined the fluorescence emission bands and the repeatability of the fuel sampling method. Since the fluorescence emission signal can vary in time, the fluorescence signal was recorded for 50 ms after 30 s from the UV source and was turned on in subsequent experiments.

**Figure 9:**
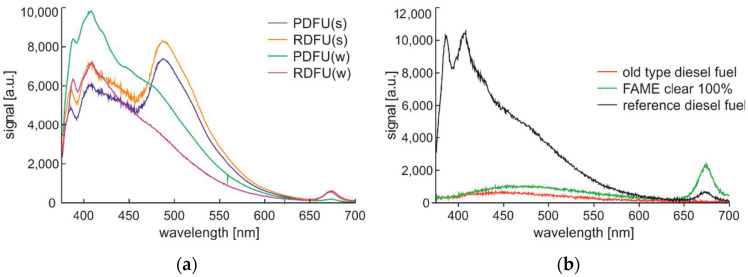
Complete spectra data of fuels examined at 0.5 min after UV source activation: (**a**) Premium diesel fuel and regular diesel fuel of winter and summer types; (**b**) old-type diesel fuel, 100% of FAME clear component, and reference diesel fuel obtained directly from a refinery.

The first study, shown in Figure 9, includes a spectral analysis of the fuels presented in Section 2.

On the left side of Figure 9, the shape of the fluorescence signal is similar for the cluster of winter and summer fuel types but not the same. In both cases, the signal is lower for the premium fuel than for the regular fuel. As lower PAH contents than regular fuel characterize premium-type fuel, see Table 2, the presented relation was expected. On the right side of Figure 9, results for nonstandard fuels are presented. The old-type diesel fuel is obtained directly from precise distillation and thus does not contain any additives. The FAME clear 100% is a bio-component without any additives. The fluorescence signal there does not show peaks characteristic of PAH components. The FAME is characterized by the highest fluorescence signals from microbial at 675 nm wavelength. Reference diesel fuel is described in comparison to other peaks by the highest low wavelength peak at 387 nm.

**Figure 10:**
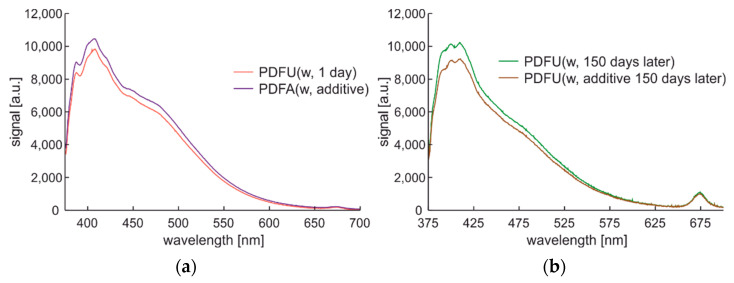
Complete spectral data of winter-type diesel stored in standard tank: (**a**) Stored one day and then examined; (**b**) unmodified premium diesel fuel stored for 150 days and then examined as PDFU (w, 150 days later) and after modification PDFU (w, additive 150 days later).

Fluorescence spectra changes introduced by the photodegradation of fuel samples can be observed in Figure 10. 

The initial characteristics of one day of photodegradation are presented in Figure 10a. Photodegradation was made here by sample storage in a standard red plastic fuel container at the windowsill for 150 days. The stored fuel sample was the further subject of modification with an additional commercial enriching additive described in Section 2. The fluorescence signal after photodegradation presented in Figure 10b shows the shifts of fluorescence peaks up to higher wavelengths. Applying an enriching additive to the degraded fuel introduces changes in the fluorescence signal level, but the shape remains the same.

**Figure 11:**
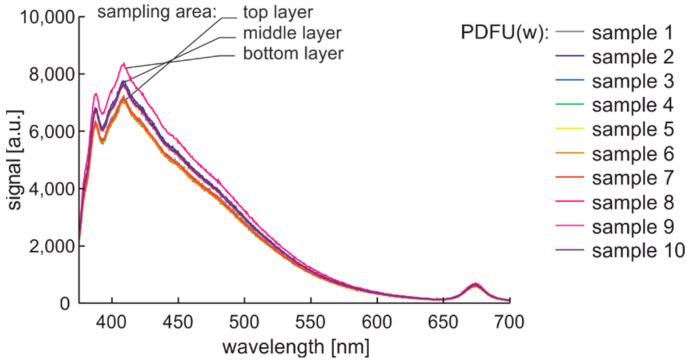
Fluorescence spectral data of premium diesel fuel in unmodified and winter type stored in a glass in a black box mounted on an anti-vibration base for a week.

The repeatability of the experiment was tested using premium fuel that was stored for a week in a glass placed in a black box mounted on an anti-vibration base. This experiment enabled the analysis of fuel sedimentation. The signals recorded for samples taken from the upper, middle, and the end of the lower layer are shown in Figure 11. Interestingly, the characteristics of the layers mentioned above coincide precisely.

Characteristics from 380 nm to 550 nm increase with the sampling depth, which indicates fluorescence particle sedimentation. Interestingly, the signal of the microorganisms is not sensitive to sedimentation, which can be explained by the fact that they were alive and mobile or had a neutral buoyancy in the oil. Thus, in the following experiment, fuels are probed from the middle layers of the samples.

### 4.2. Initial Examinations with Sequence Fluorescence Readings

Photodegradation tests use UV signals simultaneously as a degradation factor and fluorescence probe. Such tests allow flexible organization of the sampling time, but the collected characteristics are subject to varying degrees of fluorescence signal decay. Therefore, the relative change in the spectral features is essential information here.

**Figure 12:**
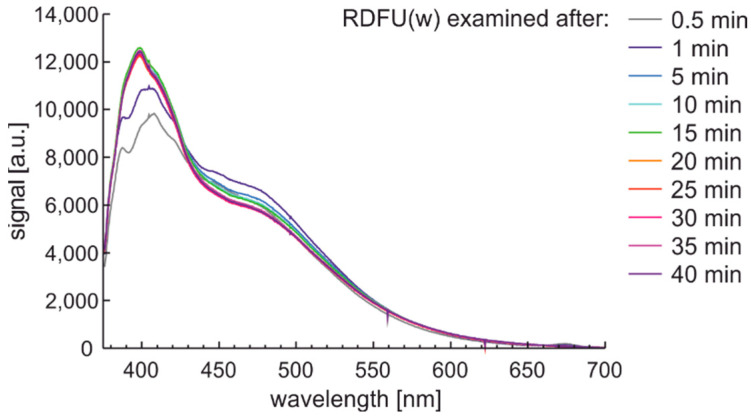
Fluorescence spectral data of regular diesel fuel unmodified and winter type examined with UV signal with constant irradiation and reading with a 5-min sequence.

Observation of the spectral changes of the signal during the photodegradation of the regular fuel of unmodified and winter type with constant irradiation and reading with a 5-min sequence is presented in Figure 12.

First, signals’ initial and rapid changes are observed at a wavelength from 380 nm to 430 nm, while slower changes are present in the 430–550 nm band. These changes seem to have minima and maxima, and the peak fluorescence signal is variable. The degradation results are visible after several min.

**Figure 13:**
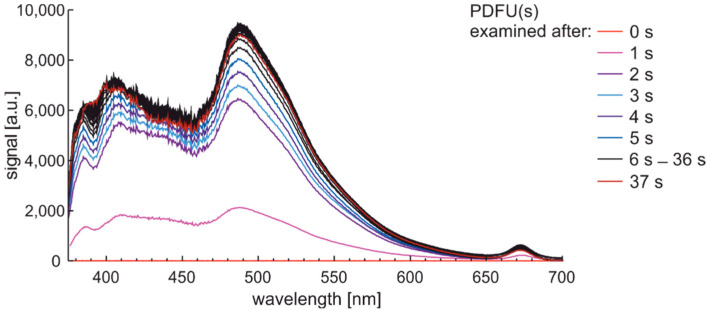
Fluorescence spectra of premium diesel fuel of unmodified and summer type while examined with a 1-s sequence of sampling.

Analogous examination with a 1-s sequence of sampling for premium diesel fuel of unmodified and summer type are presented in Figure 13.

The characteristics set from Figure 13 show that measurement setup wakes up to acquired spectral data in up to 2 s. Photoactivation of the sample requires a few seconds. Analysis of examination time leads to the conclusion that some characteristic signals of fluorescence in bandwidth from 375 nm to 600 nm need a few seconds to stabilize.

A repeatable procedure with a precise timestamp of signal acquisition is essential for diesel fuel stability testing using UV as a degradation factor and examination probe. Analyzing complete spectral data of fluorescence changes versus time is a complex task. Thus, the following sections present the investigations of time-dependent signals at a specific bandwidth. The bands are of ±5 nm width and are positioned at potentially interesting wavelengths: 380 nm, 390 nm, 410 nm, 430 nm, 490 nm, and 500 nm of the dominant hills of examined fuels, as well as the dominant 675 nm band of the microbial presence.

## 5. Operational Research of Fuel Degradation and Examination Cycles

The measurement setup enables continuous and modulated degradation of fuel samples with thermal and UV radiations and simultaneous examination of fuel degradation with UV-excited fluorescence in programmed measurement cycles. The measurement cycles used in this publication are called DCE (degradation continuous and examination) and DME (degradation modulated and examination). It should be noted that light switching requires up to 2 s, while sample photoactivation, as well as the time of sample cooling and heating, is characterized by a similar time.

### 5.1. Degradation and Examination Cycles with the Continuous Action of Degradation Factor

**Figure 14:**
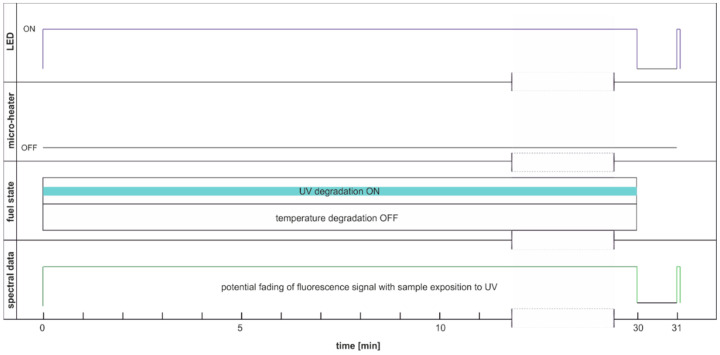
Degradation and examination cycle of fuel with UV use named DCE:UVonToff.

This section aims to exhibit the vital phenomenon of fluorescence signal fading in examining fuel using temperature and UV as continuous degradation factors. The cycle using only UV radiation for continuous degradation and studying its effects is shown in Figure 14. As the degradation factor is only UV, this cycle description is DCE:UVonToff.

**Figure 15:**
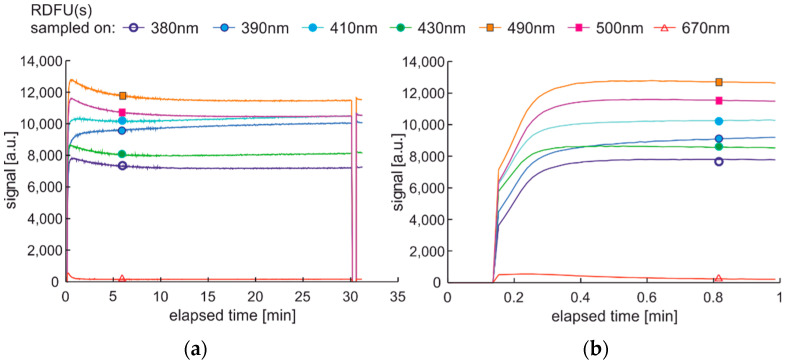
Time series of fluorescence data of regular diesel fuel in unmodified and summer type RDFU(s) examined in cycle DCE:UVonToff: (**a**) full-time series; (**b**) zoom of the initial stage.

The modification of the analyzed cycle was realized with the addition of UV switching off for 1 min at the end of 30 min degradation cycle and then again switching on. In Figure 15a, fading of fluorescence is significantly lower for exclusive photodegradation than signal changes obtained in degradation. In Figure 15b, all signals increase initially, then decrease, grow, or stabilize depending on the wavelength. Paired characteristics can be observed for pairs at 380 nm and 430 nm, 490 nm, and 500 nm. The signals at 390 nm and 410 nm differ slightly in shape, and the photoactivation of the sample is stretched over time.

**Figure 16:**
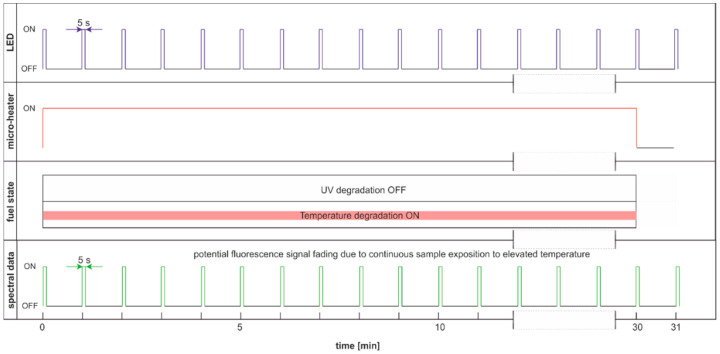
Degradation and examination cycle of fuel with UV use named DCE:UVoffTon.

**Figure 17:**
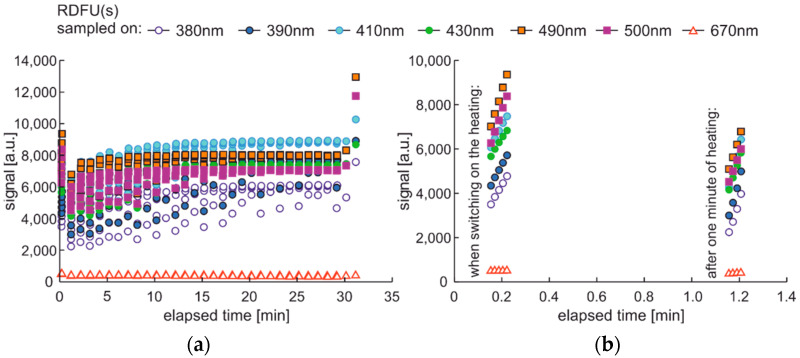
Set of time series of fluorescence data of regular diesel fuel RDFU(s) in cycle DCE:UVoffTon: (**a**) complete time series; (**b**) zoom of the initial stage.

The degradation with thermal radiation is made in the cycle named DCE:UVoffTon. It should be noted that thermal degradation requires UV light as the examination probe of degradation, as presented in Figure 16. In the analyzed case, the UV radiation is used for results readings and is on for only 5 s per 1 min. Thus, the fading of fluorescence signal due to sample exposition to UV radiation is minimal. The side result is that spectral data are measured in a one-minute cycle. Since the potential fading of fluorescence signal may still result from constant sample heating, the measurement cycle includes additional one-minute sample cooling after continuous thermal degradation at the end of a 30-min process, when further UV examination is performed. The results of the examinations are presented in Figure 17.

The thermal fading of fluorescence is visible at the beginning and at the end of the experiment. At the start of the experiment, when the sample is at room temperature, the fluorescence signals are higher than after one minute of heating—switching on UV radiation and examination of fluorescence in the following 1-s results in a local signal increase, Figure 17b. At the end of the experiment, at 31 min, the signal is much higher than for 30 min. Therefore, a minute of sample cooling and resting may be required for fluorescence signal regeneration.

The maximum fluorescence signal for the tested fuel in both degradation cycles, shown in Figure 15 and Figure 17, occurs for the 490 nm wavelength and has a similar value of about 13 thousand units. Analyzing the values of the final fluorescence signals, one can see that the results are not the same. Moreover, comparing semi-continuous data courses with data collected in packs is difficult in the analyzed case, as the maxima of fluorescence signals are outside the pack sampling rate.

**Figure 18:**
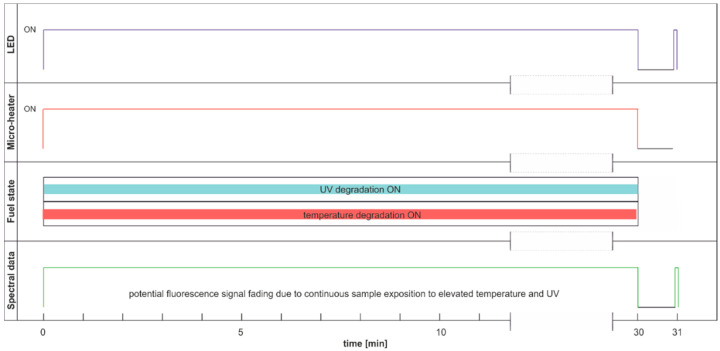
Degradation and examination cycle of fuel with UV and thermal radiation simultaneous use named DCE:UVonTon.

**Figure 19:**
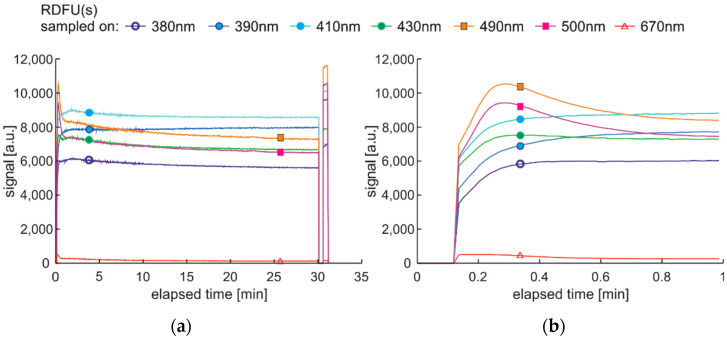
Set of time series of fluorescence data of regular diesel fuel RDFU(s) in cycle DCE:UVonTon: (**a**) full-time series; (**b**) zoom of the initial stage.

Unwanted fluorescence fading can also arise when UV and thermal radiation are used simultaneously. The corresponding cycle DCE:UVonTon is presented in Figure 18, while the results are in Figure 19.

The fluorescence fading is visible in Figure 19a. After the sample’s cooling and regeneration, the order of signals in specified wavelengths is the same as in cycle DCE:UVoffTon presented in Figure 17. At the initial 1 min of the degradation, when temperature and UV simultaneously degrade the sample, the fluorescence signals vary more rapidly and differently than in the case of UV-only use, as presented in Figure 15. The process without degradation modulation (Figure 19) shows clear signal peaks during the tests’ first minutes, but thermal fading distorted the results further. Thus, this cycle’s specific advantage is that the sample’s photoactivation is visible.

### 5.2. Degradation and Examination Cycles with the Modulated Action of Degradation Factor

In the above-presented cases, the comparison of degradation factors efficiency is loaded with uncertainty caused by potential fluorescence fading. Thus, based on the time that is required for the sample to return to the ambient temperature and fluorescence regeneration, which is less than one minute, two more measurement cycles based on double modulation are proposed. The first modulation involves excluding degradation factors after 5 min for 1 min. In this minute, the second modulation of light switching with a period of 5 s is used for degradation examination. Thus, the degradation and sample regeneration are equal in the subsequent cycles, and the action of signal fading on reading results is limited. Consequently, spectral data were collected in packets every 6 min. Thus, the proposed cycles allow the comparison of the effectiveness of degradation.

**Figure 20:**
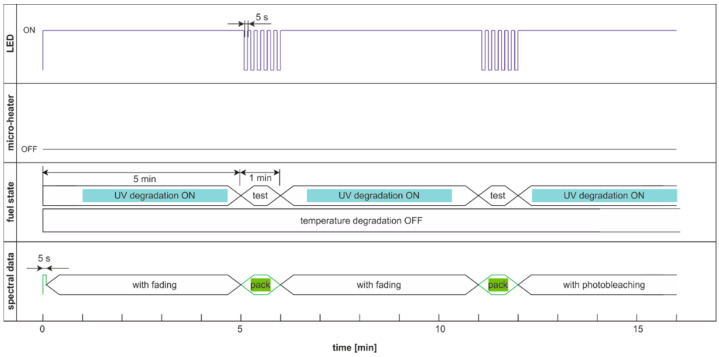
Degradation and examination cycle of fuel with double modulated UV radiation named DME:UVonToff.

**Figure 21:**
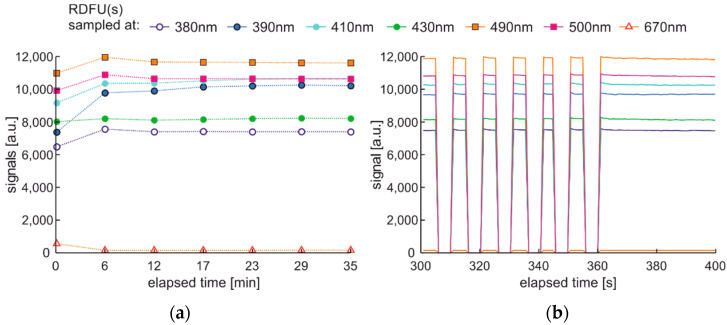
Set of time series of fluorescence data of regular diesel fuel RDFU(s) in cycle DME:UVonToff: (**a**) full-time series; (**b**) zoom of the initial pack.

The degradation and examination cycle with double-modulated UV radiation, named DME:UVonToff is presented in Figure 20, while the results are in Figure 21.

Comparing results obtained in DCE:UVonToff (Figure 15) and DME:UVonToff (Figure 21) cycles shows that due to a low sampling rate DME cycle lost information about initial signal maxima. At the same time, the characteristics after 5 min of degradation are very similar in shape and values. Analysis of the left part of Figure 21b, where the selected information pack is present, leads to a conclusion that ultra-short time fluorescence fading does not occur due to sampling irradiation.

**Figure 22:**
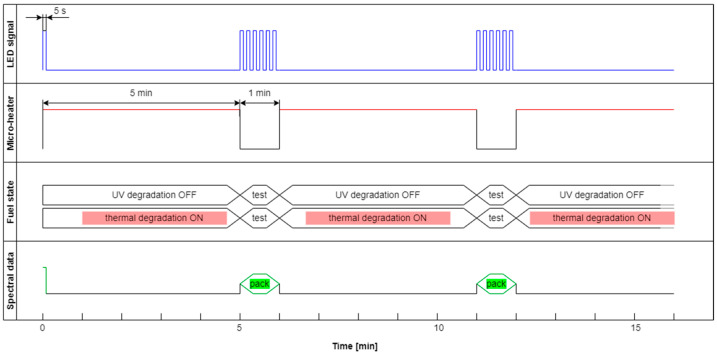
The modulated cycle of degradation and examination uses thermal radiations for degradation and modulated UV for analysis, named DME:UVoffTon.

The modulated cycle of degradation using thermal radiations for degradation and modulated UV for examination, named DME:UVoffTon, is presented in Figure 22.

**Figure 23:**
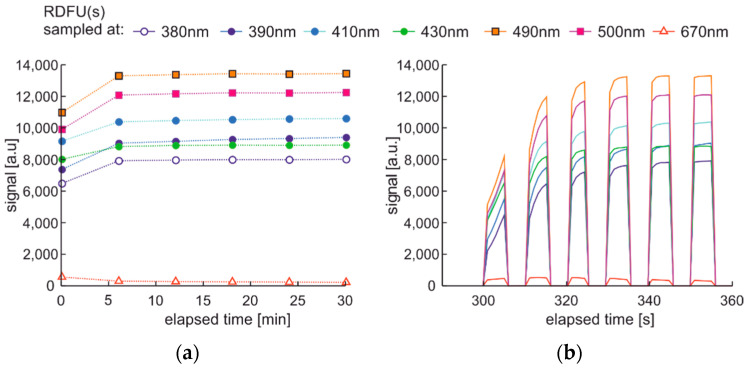
Time series of fluorescence data of regular diesel fuel RDFU(s) in cycle DME:UVoffTon: (**a**) full-time series; (**b**) zoom of the initial pack.

From Figure 23b, one can conclude that elevated temperature fading of fluorescence is reduced after 50 s from temperature switching off. Thus, the values of the last pulse in the pack are used as information. Observed variations of signals during thermal degradation (Figure 21) are similar in values to the case of UV degradation (Figure 23), but the mentioned variations are not on the same wavelengths.

**Figure 24:**
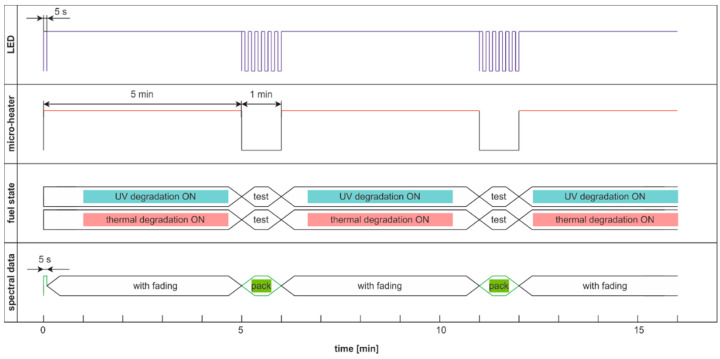
The modulated cycle of degradation and examination uses thermal and UV radiations for degradation and modulated UV for examination, named DME:UVonTon.

**Figure 25:**
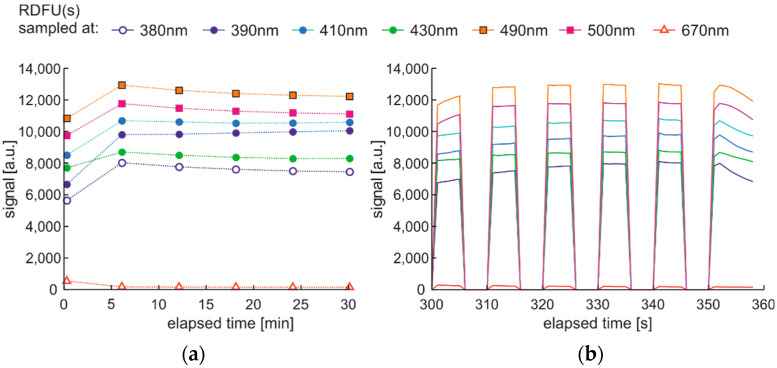
Time series of fluorescence data of regular diesel RDFU(s) in cycle DME:UVonTon: (**a**) full-time series; (**b**) zoom of the initial pack.

The modulated cycle of degradation using thermal and UV radiations for degradation and modulated UV for examination, named DME:UVonTon, is presented in Figure 24, while results are shown in Figure 25.

The degradation cycle results presented in Figure 25 are as expected. Firstly, the signal increases, then after reaching the maximum, the signal slowly decreases. The impact of the elevated temperature on signal fading is measurable but is lower than in the case when the temperature is the continuous or single degradation factor. Comparing cycles with dual thermal and UV degradation (DCE:UVonTon and DME:UVonTon) shows some limitations to both. The process with degradation modulation (Figure 25) correctly displays the course of the signals during medium-term degradation, but it lacks information about the initial course of the signal. The comparison of degradation results in modulated cycles is summarized in Table 3 and Table 4.

Results collected in Table 3 correspond to signal shifts calculated between values S(360s) and the end value of the cycle in 1800 s, S(1800s). This comparison can be attributed to a short-term test. The presented data confirm that temperature and UV degradation factors act differently on the sample. Both affect fuel stability, but the shifts of characteristics are more significant for UV degradation than for temperature. However, it should be noted that the most remarkable changes in the signal cause the simultaneous action of both factors. At the same time, the action of both factors cannot be presented as the sum of two independent actions. It should also be noted that in the mentioned short-term test, the signal values for the 670 nm band responsible for microbiological contamination are relatively small. Thus, the subsequent results in Table 4 represent an ultra-short-term test, i.e., they correspond to the shifts in the signal calculated between the values in the fifth second of S(5s) and the values in the 360 s of S(360s) of the cycle.

The ultra-short-term shifts in the 670 nm band are much more significant than in the short-time test. Thus, the interim conclusion is that microbial contamination is effectively reduced in proposed degradation cycles at their initial ultra-short-term stage. Therefore, the proposed method is not valid for testing the effect of microbiological contamination on the internal stability of the fuel.

## 6. Procedure and Experimental Results of Automotive Diesel Fuel Internal Stability Examinations

### 6.1. Procedure of Experimental Data Collection

**Figure 26:**
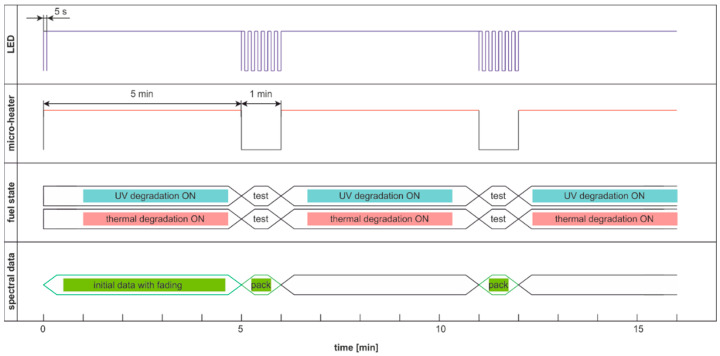
The hybrid cycle of degradation and examination uses thermal and UV radiations for degradation and modulated UV for analysis named DHE:UVonTon.

The previously presented degradation cycles have, in all cases, pairs of advantages and disadvantages. Obtained results show that both degradation factors are important to fuel internal stability; both ultra-short- and short-term data are essential, and thermal fading adversely affects the measured signals. The solution seems to be a hybrid degradation and examination cycle presented in Figure 26.

**Figure 27:**
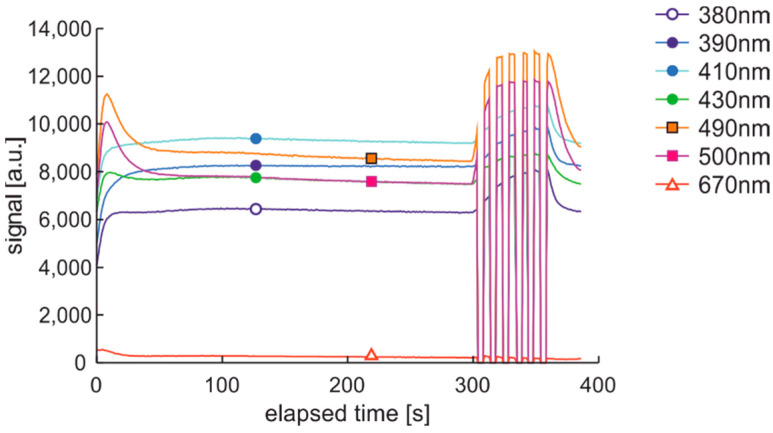
Time series of data of regular diesel fuel RDFU(s) in cycle DHE:UVonTon–zoom of the initial phase.

Obtained time series are of the expected course, as presented in Figure 27. Initial values with active thermal factor increase rapidly, as in the DCE:UVonTon cycle. The following pack of data looks very similar to DME:UVonTon cycle.

The hybrid cycle DHE:UVonTon is proposed for further tests of fuel samples with UV and temperature as degradation factors.

### 6.2. Fuels Samples Internal Stability Testing

**Figure 28:**
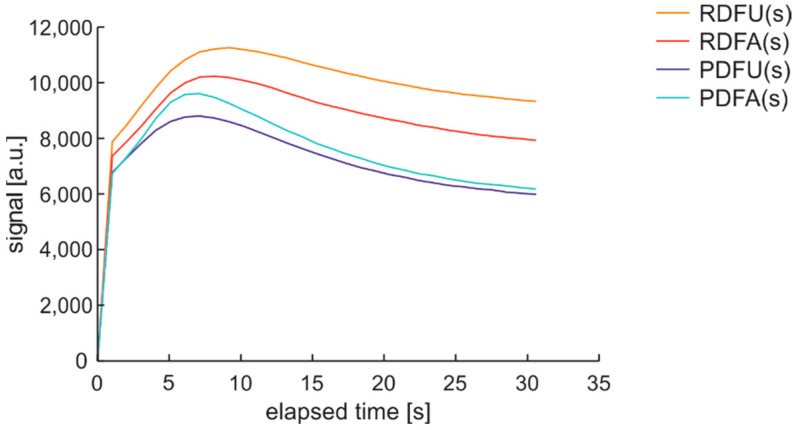
Time series at 490 nm of data of DHE:UVonTon cycle initiation.

The fuels presented in Table 2 in unmodified and modified versions were selected for analysis of hybrid degradation and examination cycle. Data of cycle initiation at 490 nm are shown in Figure 28. This wavelength chosen as maximum changes of initial signals in selected fuels were observed. It should be noted that the inclusion of degradation factors creates an unstable situation. The interpretation of signals in an unstable situation is typically different from a stable position. The maximum peak of the signal can be interpreted as the photoactivation of the medium. The signal shifts down from the maximum can be construed as a reduction in medium photoactivation. Thus, the more significant the signal drop after photoactivation, the greater the potential for fuel stability to radiation exposure.

The signal at 490 nm is higher for regular than premium fuels samples listed in Table 2, assuming that the fluorescence signal is proportional to PAH concentration when there are similar environments of the mentioned particles, and the particles are of the same distribution. It is not always the case due to differences in the petrochemical processes of fuel preparation.

Collected data of initial signal shifts calculated between maximum values S(max) and semi-stable values assumed to happen at 200 s of the cycle, S(200s), are presented in Table 5.

Since the relation of signal shifts at all selected bands is similar in the sample-to-sample comparison, the average value can be assumed to measure signal changes. This measure supposes that in the ultra-short-term examination, the internal stability is lower in the case of regular fuel than of premium fuel. Moreover, the initial internal stability was increased in both fuels by the applied additive. This agrees with the observation of Figure 3, where additive reduces color changes in the degraded fuel sample.

In stabilized situations, internal stability should be correlated to stable signals. The degradation factors should force fewer changes in signals for stable fuel than unstable. Collected data of the second part of the examination and degradation cycle corresponding to signal shifts calculated between values S(360s) and the end value of process in 1800 s, S(1800s), that are not burdened with signal fading are presented in Table 6.

The data show that medium-term internal stability is also more excellent for premium fuels than for regular fuels, but some differences in signals at specified wavelengths occur. The analysis of the internal stability of modified fuels confirms, sometimes postulated thesis, that modification with the active ingredient may result in additional, unexpected further reactions that are difficult to predict.

Most significantly, the average short-term signal shift relation between premium and regular fuel is 1.07 (Table 6), while the ratio between PAH concentrations (see Table 2) is 1.08. Thus, the internal stability of diesel fuels can be related to PAH concentration.

**Figure 29:**
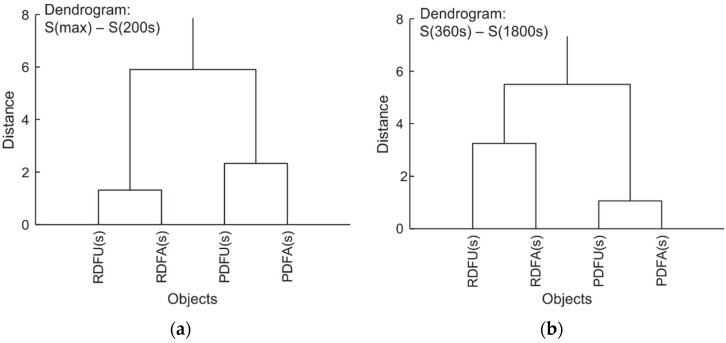
Dendrograms of wavelength shifts: (**a**) data from Table 5; (**b**) data from Table 6.

A summary analysis of the experiment can be made with cluster analysis. The corresponding dendrograms for wavelength shifts of data collected in Table 3 and Table 4 are presented in Figure 29.

Cluster analysis shows that premium fuel remains premium during degradation regardless of the permissible modifications. The same applies to standard fuel. It can therefore be stated that the proposed measurement procedure does not introduce unexpected errors. Thus, comparing the results of signal shifts for different fuels can serve as a differential classification for the internal stability of the fuel under testing. The fuel is more stable when signals of shifts S(360s)–S(1800s) are lower than in the compared sample. Fuel also is expected to be more stable when signals shift S(max)–S(200s) of initial degradation is more significant than in the referred sample.

## 7. Conclusions

In the presence of oxygen, automotive diesel fuel degrades significantly. However, automotive diesel fuel samples without contact with oxygen can also degrade. We presented the results of the examination of internal fuel stability with the separate and simultaneous use of two degradation factors of thermal and UV radiations as they can be applied without physical contact and the use of any chemical reagents.

We have shown that such examination is possible when fuel is positioned in a glass capillary optrode so that the outer sections of the fuel fill the capillary, preventing the middle section from direct exposure to the atmosphere. We have shown that the continuous temperature degradation factor results in significant fluorescence fading and can prevent proper examination. Thus, we conclude that realizing degradation with two degradation factors, such as temperature and UV radiation, is not a trivial task.

From the point of view of the internal stability of the automotive diesel fuel, microbiological contaminants are positioned at the fuel–oxygen–water interface, so they are present inside the sample in limited concentrations. Moreover, UV radiation has a deadly effect on microorganisms. Thus, fast signal fading from live microorganisms is a side effect of the analyzed method.

Examination of different fuels with continuous UV degradation leads us to the conclusion that specific characteristics of fluorescence may differ significantly. Moreover, fluorescence peaks during degradation may shift in value and wavelength. Thus, signal shifts at specified wavelengths are not a good measure of fuel stability, and the signal shifts should be measured in bands. In examined cases, the bands with 10 nm fulfill the task. It should also be noted that the fluorescence signal restoring from fading in explored cases is at the rank of 50 s while the time stabilization of the sample in heating and cooling is less than 15 s. Additionally, the time of the UV source activation is not to be omitted.

Realized operational research of degradation cycles leads us to a hybrid degradation cycle that is a mixture of continuous 300 s ultra-short-time section and modulated 1500 s short-time degradation. This way, the proper reading of degradation results is possible. Measurement setup enables constant access to information in an ultra-short period of 300 s and the semi-continuous reading of short-term degradation. This hybrid cycle contains complete information on the degradation. Using it allows proper classification of the internal fuel stability with photoactivation time as its ultra-fast signal shifts as well as its short-term signal shifts in the range from 360 s of degradation to the end value of the cycle in 1800 s.

Thus, well-defined time synchronization of degradation action and its results reading is required, and the realization of examination involves quite a complex measurement setup. Again, these are costs of any chemical used, and no initial sample is prepared for internal stability testing of automotive diesel fuel.

The short list of the practical side of the investigation is as follows:

- Temperature and UV degradation factors act differently on the sample.
- At the same time, the action of both factors cannot be presented as the sum of two independent actions.
- The UV degradation factor acts stronger than temperature, but both factors simultaneously used are the key to the speed and effectiveness of examinations.
- The internal stability of automotive diesel fuel is related to the presence of unsaturated components and can be characterized by fluorescence signal shifts.
- The internal stability of diesel fuel is more related to the proposed method than to the standard measurement of oxidative stability.
- The proposed procedure and setup enable the comparison of different fuels’ internal stability using reference data. This comparison allows us to point to more internally stable fuel.

Thus, as the main practical result of the research, we propose the differential classification of the internal stability of fuels that may be included in future commercial specifications and fuel parameters guarantees. Future examinations can be oriented toward validating the proposed method.

## Figures and Tables

**Table 1:** Example PAHs concentration in diesel fuel and crude oil and characteristics parameters.

Parameter	Naphthalene	Methylnaphthalene	Phenanthrene	Pyrene	Perylene	Benzo[a]pyrene
concentration in diesel fuel [mg/kg]	1100	5910	1250	82	0	0
concentration in crude oil [mg/kg]	42	109	75	13	12	4
Ring number	2	2	3	4	5	5
Boiling point [°C]	217	240	338	394	467	495
Appearance	white crystalline volatile solid	colorless liquid	colorless monoclinic crystals	colorless solid	yellow to colorless solid	Pale yellow crystals

**Table 2:** Measured parameters of summer fuels used in this study.

Fuel Type	Cetane Number (ISO 5165)	Fuel Stability (EN 15751) [h]	Water Volume [mg/kg]	PAH [% (m/m)]	FAME (EN 14078) [% (*v/v*)]
Premium PDFU(s)	55	20	103	1.85	0.05
Regular RDFU(s)	52	20	105	2.00	7

**Table 3:** Signal shifts S(360s)-S(1800s) obtained during the degradation of regular diesel RDFU(s) during the DME cycle.

Cycle Type	380 nm	390 nm	410 nm	430 nm	490 nm	500 nm	670 nm
DME:UVonToff	161	−436	−291	−10	343	252	−9
DME:UVoffTon	−90	−359	−218	−99	−142	−155	65
DME:UVonTon	567	−250	103	398	716	641	24

**Table 4:** Results in band 670 nm of ultra-short-term test for RDFU(s) degradation in the DME cycle.

Cycle Type	[S(5s)–S(360s)] at 670 nm
DME:UVonToff	406
DME:UVoffTon	260
DME:UVonTon	362

**Table 5:** Signals shifts S(max)–S(200s) of initial degradation in DHE:UVonTon cycle.

Fuel Type	380 nm	390 nm	410 nm	430 nm	490 nm	500 nm	670 nm	Avg
RDFU(s)	76	49	124	321	2663	2457	287	854
RDFA(s)	101	114	248	838	3093	2844	271	1073
PDFU(s)	1485	313	1273	1752	3488	3103	139	1650
PDFA(s)	1999	977	1995	2194	3972	3573	144	2122

**Table 6:** Signals shifts S(360s)–S(1800s) of the modulated part of degradation DHE:UVonTon cycle.

Fuel Type	380 nm	390 nm	410 nm	430 nm	490 nm	500 nm	670 nm	Avg
RDFU(s)	567	−250	103	398	716	641	23	314
RDFA(s)	502	529	690	679	1131	1026	49	658
PDFU(s)	1116	683	264	7	52	−47	−25	293
PDFA(s)	1182	667	395	22	−273	−342	−51	228

## Data Availability

Not applicable.
